# Retinal Hæmorrhages in Fracture of the Skull

**Published:** 1903-04-25

**Authors:** 


### RETINAL HÆMORRHAGES IN FRACTURE OF THE SKULL.

As the result of some interesting observations on the eyes of persons who had died from fracture of the base of the skull or from intracranial hæmorrhage involving the subarachnoid space without fracture, Dr. Fleming[1] draws some conclusions which, if established as correct in the light of further inquiry, should make an important addition to our knowledge of these conditions. He found that, on examining the eyes of persons who had died from fracture of the base of the skull in which there had been considerable effusion of blood into the subarachnoid space, retinal hæmorrhages were usually present, and that where the subarachnoid effusion was unilateral the retinal hæmorrhages were as a rule limited to the same side. The hæmorrhages in the retina were usually massive, or of irregular outline, and were situated at some distance from the optic disc. In cases where the subarachnoid effusion comes on gradually no hæmorrhages occur in the retina. Dr. Fleming mentions twelve cases of fractured skull on which he bases his conclusions. Eleven were cases of fractured base and in one there was a fracture of the squamous part of one temporal bone. He divides these cases into three groups: (1) Five cases in which the subarachnoid effusion was mostly unilateral and retinal hæmorrhages were present but confined to the eye on the same side; (2) two cases in which the effusion was almost equally marked on the two sides, and in which there were retinal hæmorrhages in both eyes; (3) five cases in which no retinal hæmorrhages were found. In all these, with two exceptions, there was but little subarachnoid hæmorrhage, or an effusion of apparently slow onset, and in one of these pressure seems to have interfered with the passage of blood into the intersheath spaces of the optic nerve. In order to exclude the fallacy as to the retinal hæmorrhages being due to the concussion or other conditions associated with fracture of the skull, four cases of subarachnoid effusion not due to fracture were examined. In three of these retinal hæmorrhages were found. In one they were present in both eyes, in the other two they were confined to the side of the chief subarachnoid effusion alone. Dr. Fleming thinks that it is reasonable to conclude that a subarachnoid hæmorrhage, if sufficiently rapid in its development, will cause retinal hæmorrhages, and that if the effusion is unilateral the hæmorrhages will be confined mostly to the affected side.

[1] Edinburgh Medical Journal, April.

